# Supplementary material for: Breaking epigenetic shackles: targeting ARID1A methylation and the PI3K/AKT/mTOR-PD-L1 axis to overcome immune escape in gastric cancer
Source: PeerJ. 2025 Nov 6;13:e20251. doi: 10.7717/peerj.20251 (PMC12596888; doi:10.7717/peerj.20251)
Supplement: Supplemental Information 1 [file peerj-13-20251-s001.doc]

**Supplementary data**


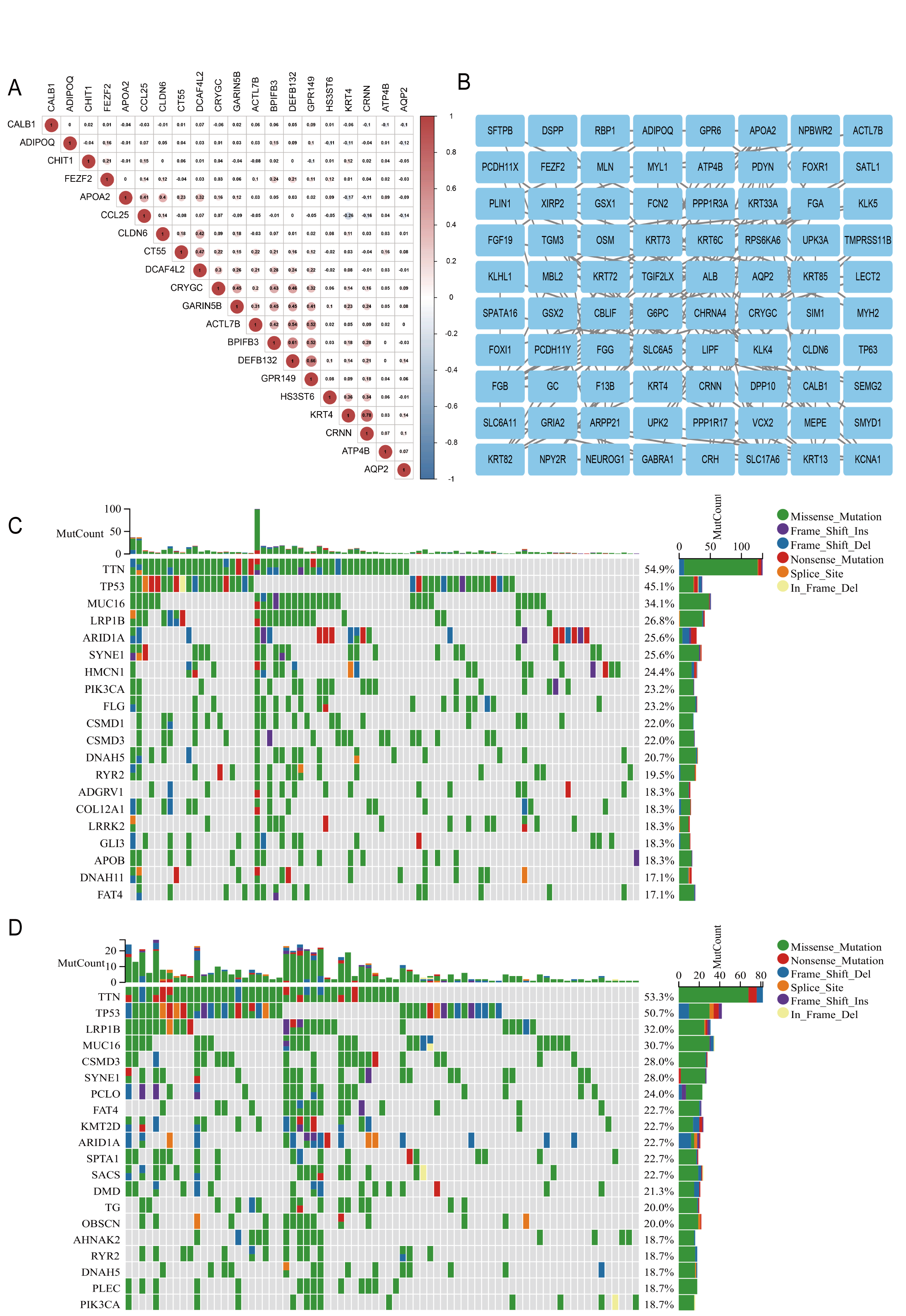


**Figure S1. Multi-Omic Characterization of Methylation-Driven Alterations in Gastric Cancer**

1. Correlation matrix of the top 10 upregulated and downregulated MeDEGs.
2. Protein-protein interaction (PPI) network reconstructed from STRING database and visualized in Cytoscape.
3. Top mutated genes in the ARID1A hypermethylated cohort: TTN (54.9%), TP53 (45.1%), MUC16 (34.1%), LRP1B (26.8%), and ARID1A (25.6%).
4. Mutation spectrum of the hypomethylated subgroup: TTN (53.3%), TP53 (50.7%), LRP1B
5. (32.0%), MUC16 (30.7%), and CSMD3 (28.0%).


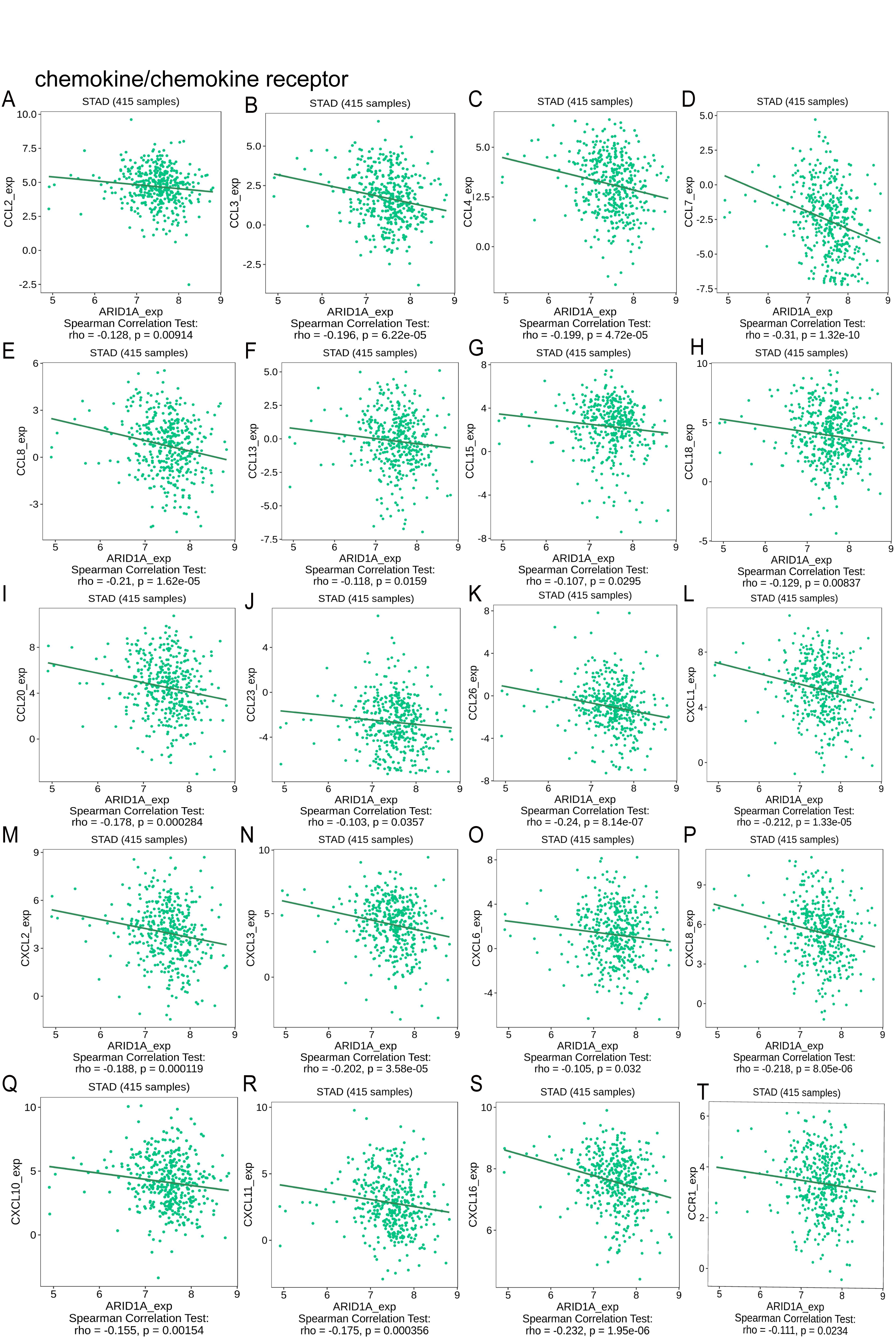


**Figure S2. Comprehensive negative correlations between ARID1A expression and pro-inflammatory chemokine/chemokine receptor levels in gastric cancer, as identified through the TISIDB database.**

(A-T) Scatter plots with fitted regression lines illustrating the significant inverse associations between ARID1A mRNA expression and the transcript levels of various chemokine ligands and receptors, as analyzed through the TISIDB database. Each plot is annotated with the Pearson correlation coefficient (r) and the corresponding p-value.

**
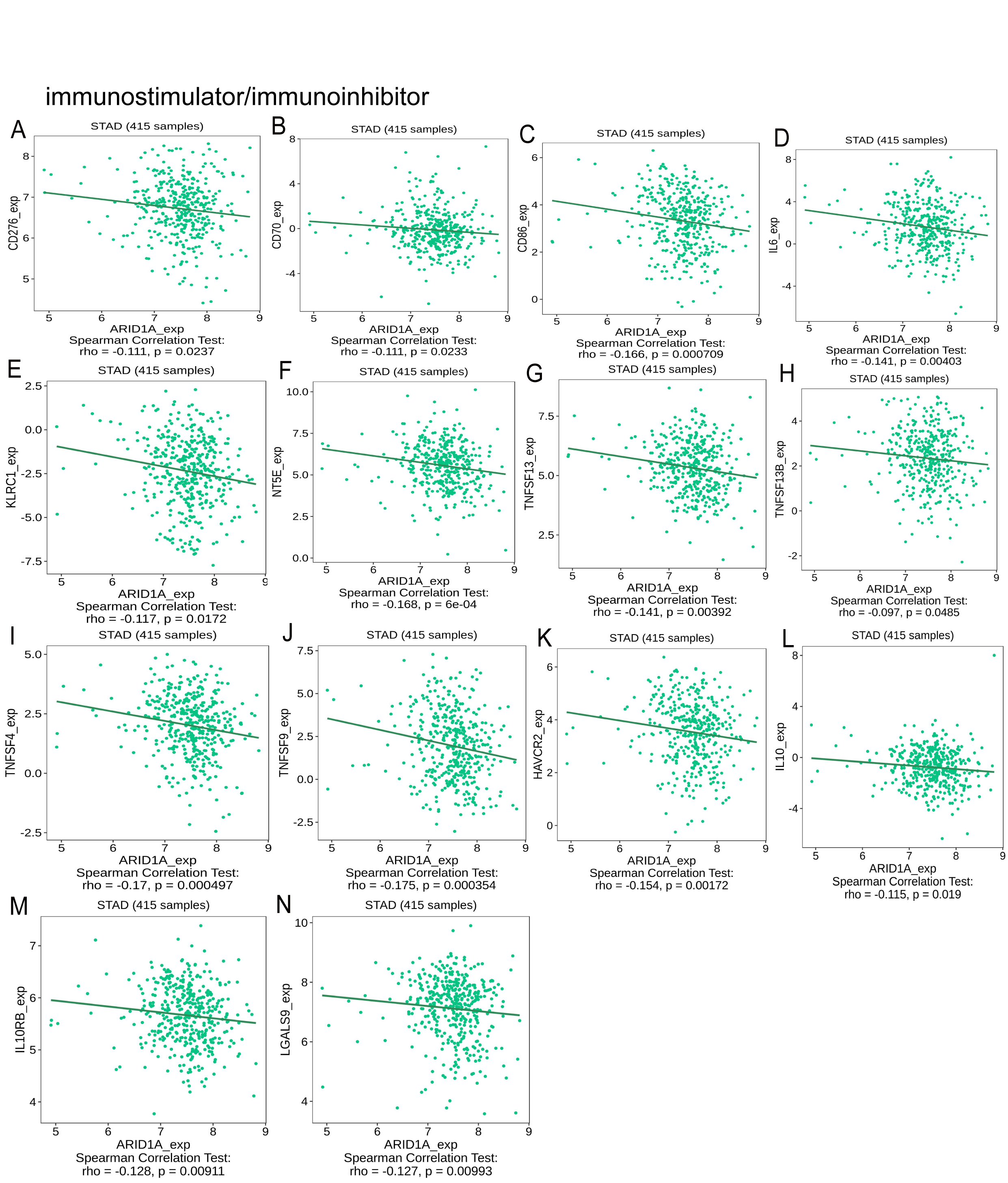
**

**Figure S3. ARID1A expression is inversely correlated with a panel of immunomodulatory molecules in gastric cancer, as identified through the TISIDB database.**

(A-N) Scatter plots illustrating the significant negative correlations between ARID1A mRNA levels and various immunostimulatory and immunoinhibitory molecules. Select correlations are annotated with Pearson correlation coefficient (r) and p-value.


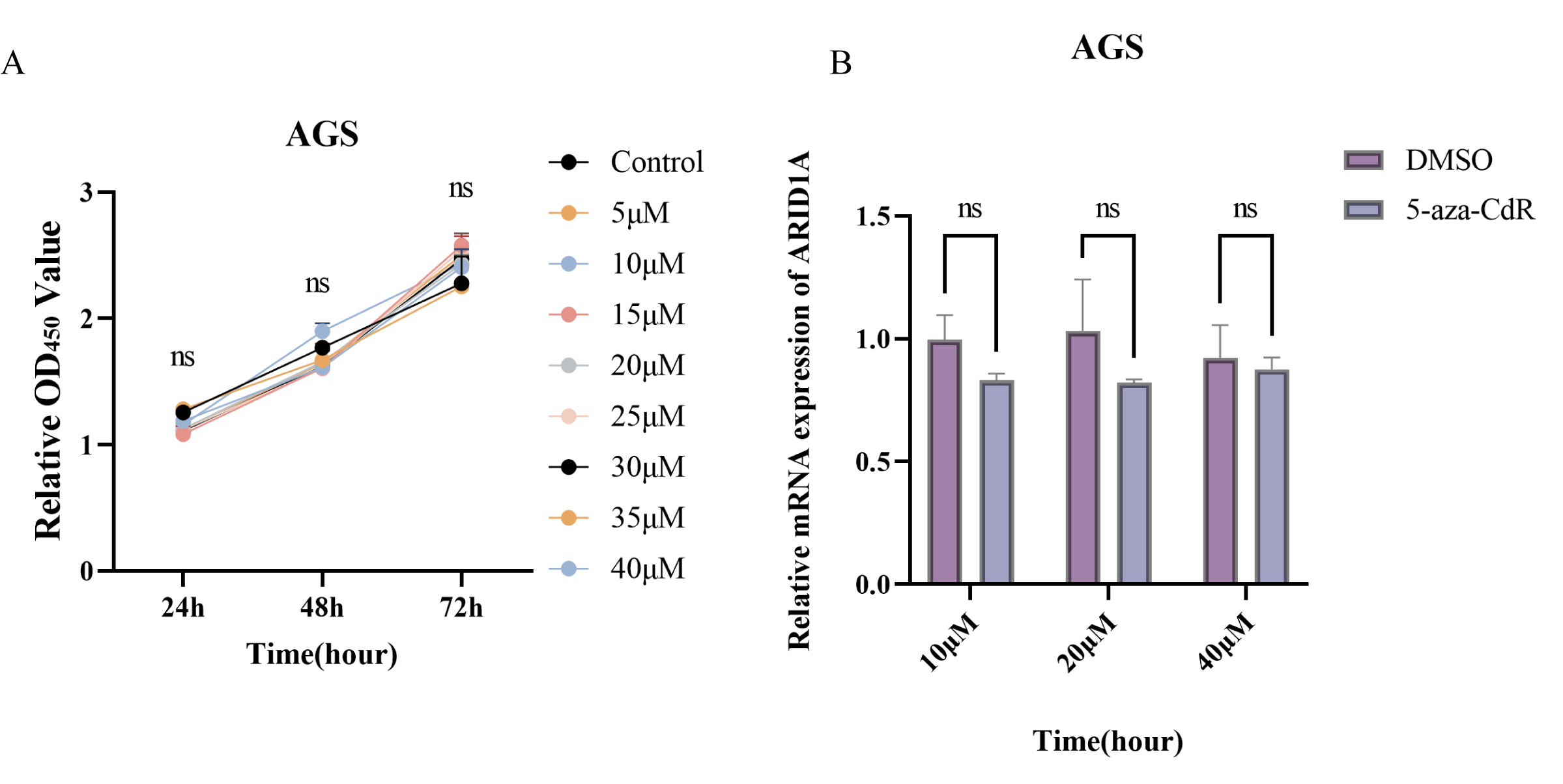


**Figure S4. Efficacy of 5-Aza-CdR in AGS Cells**

A. Dose-response proliferation curves of AGS cells treated with 5-aza-CdR. CCK-8 assay assessing AGS cell viability across a 0–40 μM concentration gradient of 5-aza-CdR, demonstrating minimal proliferative suppression even at maximal dosage.

B. Quantitative PCR (qPCR) analysis of ARID1A expression in AGS cells treated with 10, 20, or 40μM 5-aza-CdR for 48 hours, demonstrating no significant upregulation compared to untreated controls.


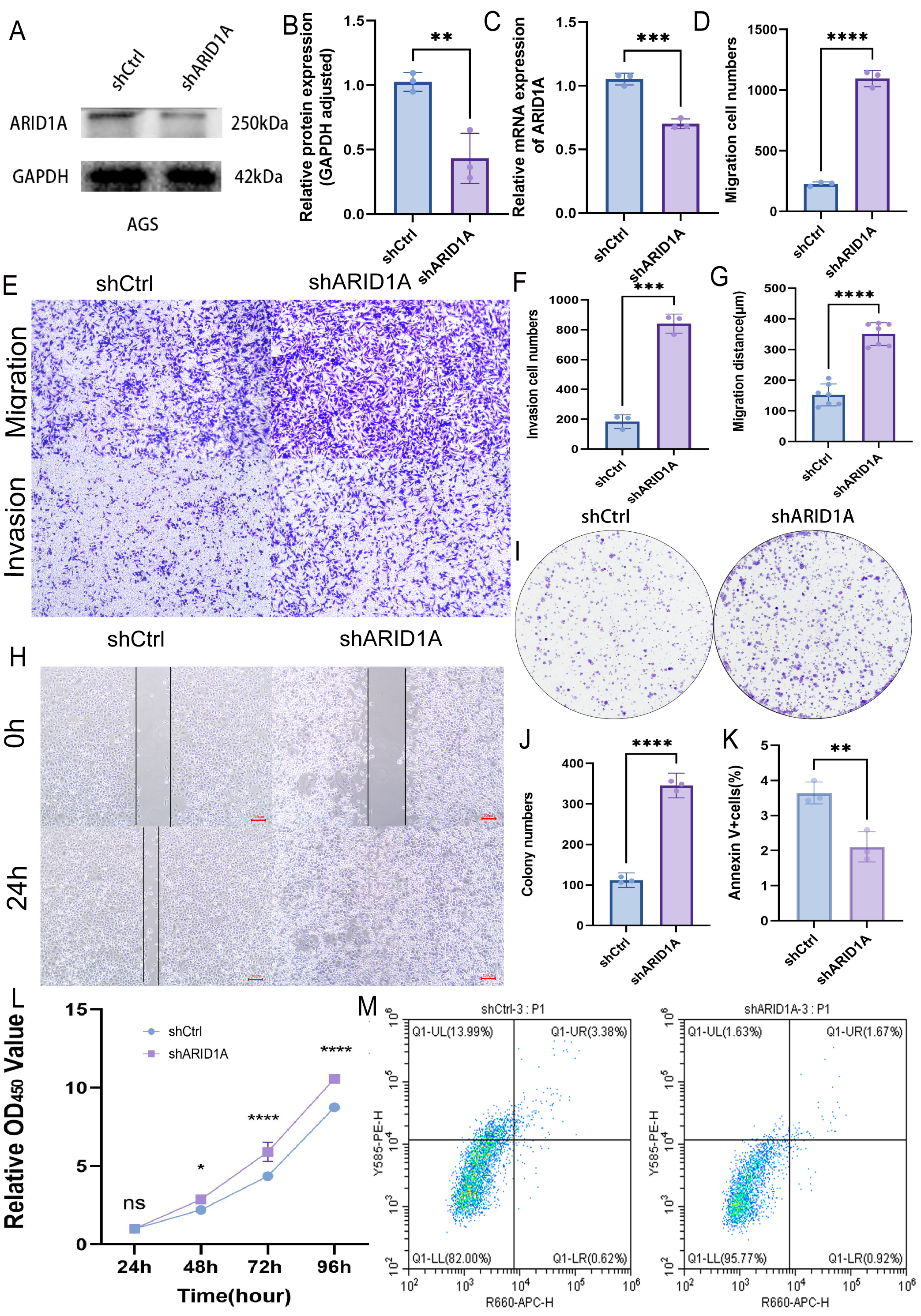


**Figure S5. ARID1A knockdown promotes malignant phenotypes in gastric cancer AGS cells.**

(A-C) Validation of ARID1A knockdown efficiency in AGS cells via Western blot (protein level) and qPCR (transcript level).

(D-F) Transwell invasion and migration assays (10x magnification) demonstrating enhanced metastatic potential in ARID1A-deficient AGS cells, visualized by crystal violet staining and analyzed using GraphPad Prism 9.0.

(G-H) Wound-healing assays quantifying accelerated migration rates in ARID1A-silenced AGS cells.

(I-J) Colony formation assays confirming enhanced clonogenic survival in ARID1A-depleted AGS cells.

(L) CCK-8 proliferation assays showing increased growth kinetics post-ARID1A knockdown.

(K, M) Flow cytometry analysis of apoptosis resistance in ARID1A-silenced AGS cells. Quantification data analyzed using GraphPad Prism 9.0 (* p < 0.05, **p < 0.01, ***p < 0.001, ****p < 0.0001)


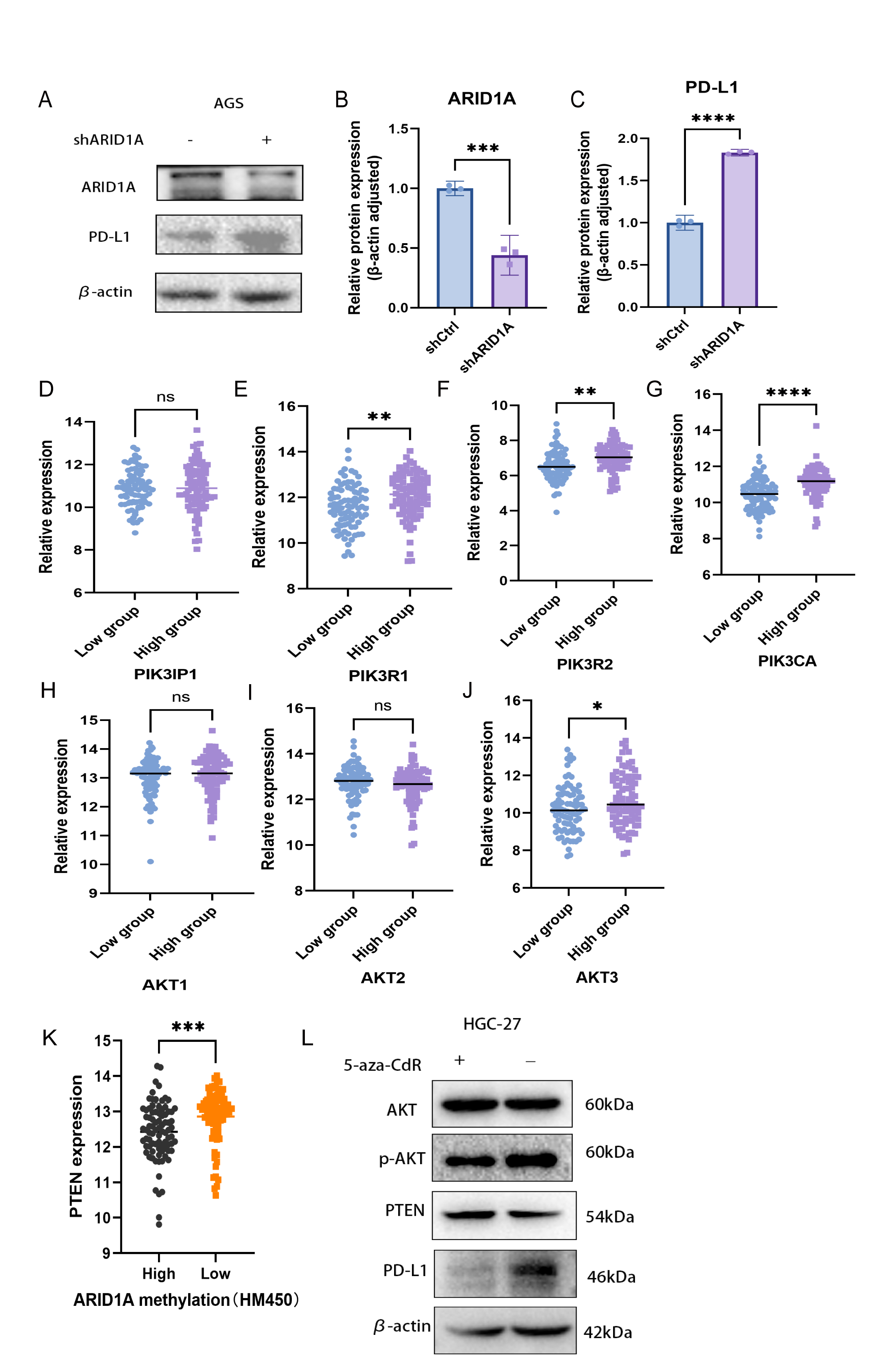


**Figure S6. Supplementary evidence supporting the epigenetic-immune regulatory axis.**
(A-C) Western blot analysis and quantification of ARID1A and PD-L1 expressions in AGS cells.

(D-J) The mRNA expression of PI3K/AKT signaling pathway genes in gastric cancer patients from TCGA cohorts stratified by ARID1A promoter methylation status.

(K) Inverse correlation between ARID1A promoter methylation (cg05445839) and PTEN expression in gastric cancer (*p* = 0.0005).

(L) 5-Aza-CdR treatment of HGC-27 cells markedly increased PTEN protein levels while suppressing p-AKT and PD-L1 expression.
