# Supplementary material for: Breaking epigenetic shackles: targeting ARID1A methylation and the PI3K/AKT/mTOR-PD-L1 axis to overcome immune escape in gastric cancer
Source: PeerJ. 2025 Nov 6;13:e20251. doi: 10.7717/peerj.20251 (PMC12596888; doi:10.7717/peerj.20251)
Supplement: Supplemental Information 7 [file peerj-13-20251-s007.pdf]

1 **Supplemental Table 1.** MIQE 2.0 checklist for authors, reviewers and editors

	PROVIDED [a]	DESCRIPTION/JUSTIFICATION [b]
**1. REAGENT PREPARATION**		
Bioinformatics tools and versions and settings used to design assays	Y	Primer-BLAST (NCBI v4.0) was used to design assays with parameters: Tm 58–62°C, exon-spanning amplicons (80–200 bp), and specificity validated agains RefSeq mRNA (2023).
Official gene symbol, species and sequence accession number	Y	ARID1A (Homo sapiens), NM_006015.6（mRNA）
Location of amplicon	Y	The ARID1A amplicon spans the exon 3–4 junction (intron-spanning design, 130 bp in length) in transcript NM_006015.6, avoiding genomic DNA amplification.
Amplicon length		130 bp
Primer and probe sequences[c]	Y	p.9, line 179-182
Location and identity of any modifications	N	Not applicable
Manufacturer of oligonucleotides	Y	Sangon Biotech (Shanghai) Co., Ltd., China
Details of optimization performed	N	Not applicable
**2. SAMPLE PREPARATION**		
Detailed description of sample types and numbers	Y	p.8, line 160-169
Sampling procedure (including time to storage)	Y	p.9, line 171-172
Sample aliquoting, storage conditions and duration	Y	stored at -80°C(≤1 months)
Description of extraction method including amount of sample processed	Y	p.9, line 171-175
Source and amount of spike-in nucleic acids added	N	No spike-in nucleic acids were added (the experiment relies on the endogenous control gene β-actin for normalization).
Volume of elution buffer used to elute/resuspend nucleic acids	Y	30–50 μL of RNase-free ddH_2O
Number of extraction replicates	Y	n>3
Extraction blanks and percent yield included	Y	Yield was not explicitly calculated, but RNA concentration (Qubit: 120 ng/μL) and purity (OD260/280 = 2.0) met the requirements for qPCR.
Method to evaluate quality and quantity of nucleic acids	Y	NanoDrop 2000 (A260/A280:1.8-2.1)
Storage conditions: temperature, concentration, duration, buffer, aliquots	Y	Stored at -80°C in RNase-free ddH_2O (50 ng/μL), aliquoted into 50 μL/tube, stable for ≤12 months.
Clear description of dilution steps used to prepare working template solution	Y	p.9, line 171-172
Template modification (digestion, sonication, pre-amplification, DNAse treatment etc.)	N	No template modifications (DNase, sonication, digestion, or pre-amplification) were performed; genomic DNA was removed by the reagent's precipitation step, validated by No-RT controls (Cq ≥40).

Purification after modification	N	No additional purification was required after RNA extraction, as genomic DNA and contaminants were removed during the RNA-easy Isolation Reagent protocol via chemical precipitation (isopropanol/ethanol washes). RNA was eluted in RNase-free water, and purity was validated by OD260/280 (1.8–2.0) and No-RT controls (Cq ⩾40).
**3. REVERSE TRANSCRIPTION[d]**		
cDNA priming method and primer concentration	Y	Oligo(dT) 100μM
One or two-step protocol (include reaction details for two-step)	Y	p.9, line 173-175 (Two-step RT-qPCR)
Amount of RNA used per reaction	Y	p.9, line 174
Detailed reaction components and conditions	Y	p.9, line 173-175
Estimated copies measured with and without addition of RT[e]	Y	Estimated copies with RT (+RTe) were 1.2×10^4 copies/μL for ARID1A and 2.5×10^5 copies/μL for β-actin (standard curve method), while no amplification was detected without RT (-RTe) (Ct ⩾40 cycles; detection limit: 10 copies/μL).
Manufacturer of reagents, catalog number and lot number	Y	p.9, line 173-175(18090050, 3238857)
Storage of cDNA: temperature, concentration/dilution, duration, buffer, aliquots	Y	Stored at -80°C in RNase-free ddH_2O (10 ng/μL), aliquoted into 20 μL/tube, stable for ⩽12 months.
**4. qPCR PROTOCOL**		
Template treatment (initial heating or chemical denaturation)	Y	p.9, line 182-184
Primer and probe concentration in the reaction and source	Y	10 μM(Sangon Biotech (Shanghai) Co., Ltd., China)
Polymerase identity and concentration, $Mg^{2+}$and dNTP concentrations[f]	Y	Polymerase (Vazyme Q711-02).<br>Mg^{2+}: 3 mM (as per manufacturer's formulation).<br>dNTPs: 0.2 mM each dNTP (pre-mixed in master mix).
Buffer/kit (manufacturer, catalog number and lot number)	Y	p.9, line 176-178(Vazyme ,Cat:Q711-02,Lot:7E2830G4)
Complete thermocycling parameters including reaction volume	Y	p.9, line 182-184
Manufacturer and type of qPCR instrument	Y	p.9, line 176-178
**5. DATA ANALYSIS**		
Storage and submission of raw fluorescence data using RDES[g] or RDML[h]	Y	Supplemental Files:QPCR.xlsx
Identity of standards (synthetic, plasmid, genomic, IVT[i], mRNA etc.) and method of quantification	Y	2^(-ΔΔCt) method
Method of baseline correction and Cq determination	Y	Automatically performed by Bio-Rad CFX Maestro software ; Cq values were derived from the intersection of the fluorescence curve with the threshold line.
qPCR analysis program (source, version)	Y	QuantStudio™ Design & Analysis Software(Thermo Fisher Scientific (USA),v1.5.1)

Details of positive and negative controls	Y	Positive Controls β-actin (ACTB): Ct <25 (all samples).<br>Negative controls (No-Template Control (NTC)) .
Frequency and Cq of negative controls	Y	NTC showed undetectable amplification (Cq ≥40).
Examples of positive and negative results	Y	Supplemental Files:QPCR.xlsx
PCR efficiency estimation and method for its determination	Y	Standard Curve
Method of target quantity calculation[j]	Y	2^(-ΔΔCt) method
Description of replicates	Y	Supplemental Files:QPCR.xlsx(n>3)
Repeatability (intra-experiment variation)	Y	Supplemental Files:QPCR.xlsx
Reproducibility (inter-experiment/user/lab etc. variation)	Y	Intra-Experiment CV <5% (n=3 technical replicates)<br>Inter-Experiment ΔCt<1.0 (tested across 2 independent runs)<br>Supplemental Files:QPCR.xlsx
Limit of detection calculated?	N	Not applicable
Dynamic range (limits of quantification)	N	Not applicable
Method of validation of reference genes	Y	ΔCt
Description of normalization method / calculation of normalized expression	Y	2^(-ΔΔCt)
Statistical methods used for analysis	Y	p.9, line 184-185
Choice of significance level and calculation of statistical power	Y	P<0.05
Specificity (when measuring rare mutations, pathogen sequences etc.)	N	Not applicable

[a]Authors should insert “Yes” or “No”.

[b] If “Yes”, specify the location of the information in the article or include the information here. If “No”, outline the rationale for omission.

[c]Disclosure of the primer and probe sequences is highly desirable and strongly encouraged. However, when commercial pre-designed assay vendors do not release this information, assay context sequences must be submitted.

[d]This section and parts of Section 5 may not apply depending on the experiment.

[e]Assessing the absence of DNA using a no RT assay (or where RT has been inactivated) is important when first extracting RNA. Once the sample has been validated as DNA-free, inclusion of a no-RT control is desirable, but no longer essential.

[f]Details of reaction components are highly desirable, however not always provided by commercial vendors. Inclusion of reagent manufacturer, catalog and batch number as well as assay context sequences is necessary where component reagent details are not available.

[g]Real-time PCR Data Essential Spreadsheet Format (1).

[h]Real-Time PCR Data Markup Language (2).

[i]*in vitro* transcribed.

[j]Efficiency-corrected target quantity calculation is necessary.

References

1. Untergasser A, Hellemans J, Pfaffl MW, et al. Disclosing quantitative RT-PCR raw data during manuscript submission: a call for action. Molecular Oncology 2023; 17:713–17.
2. Lefever S et al. RDML: structured language and reporting guidelines for real-time quantitative PCR data. Nucleic Acids Res 2009; 37:2065–69.
