# Supplementary material for: Breaking epigenetic shackles: targeting ARID1A methylation and the PI3K/AKT/mTOR-PD-L1 axis to overcome immune escape in gastric cancer
Source: PeerJ. 2025 Nov 6;13:e20251. doi: 10.7717/peerj.20251 (PMC12596888; doi:10.7717/peerj.20251)

1.Primary Western Blot data

Figure7C

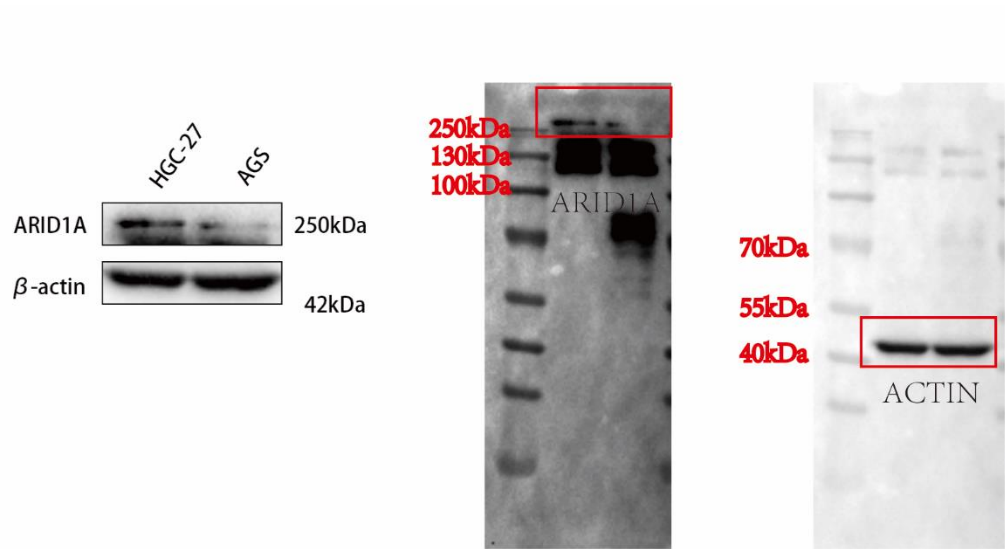

Figure7I

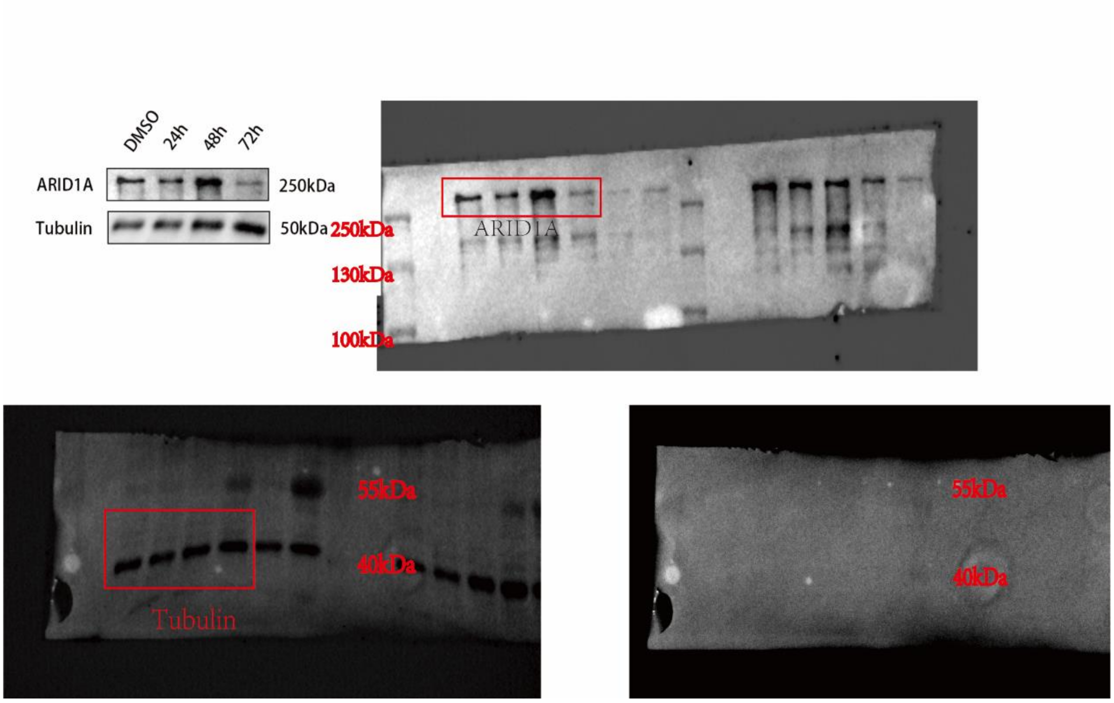

Figure8A& FigureS5A

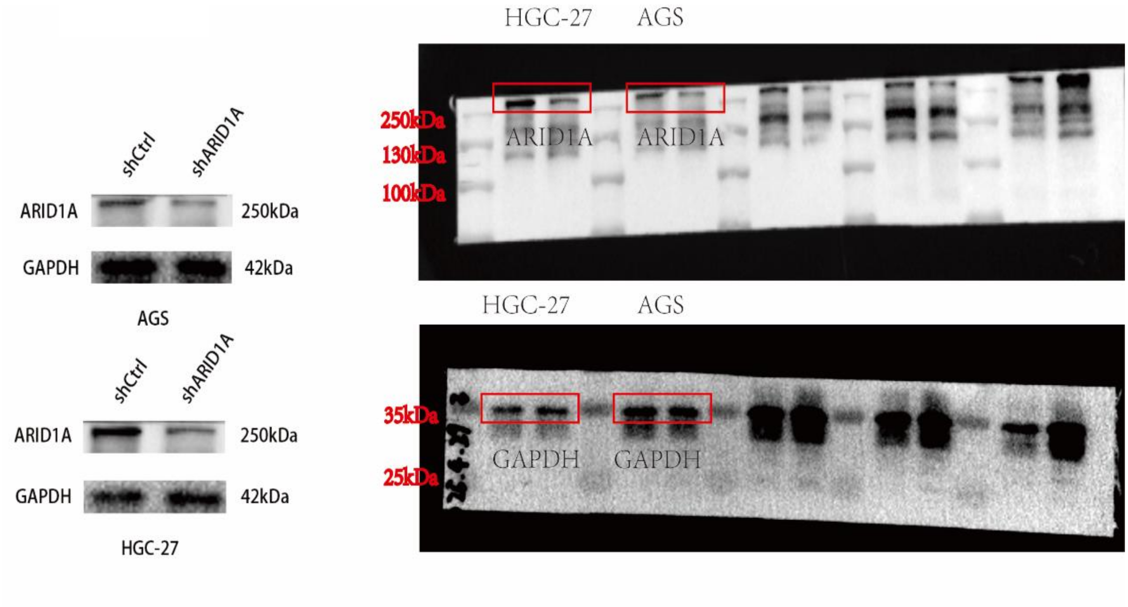

Figure10A& FigureS6A

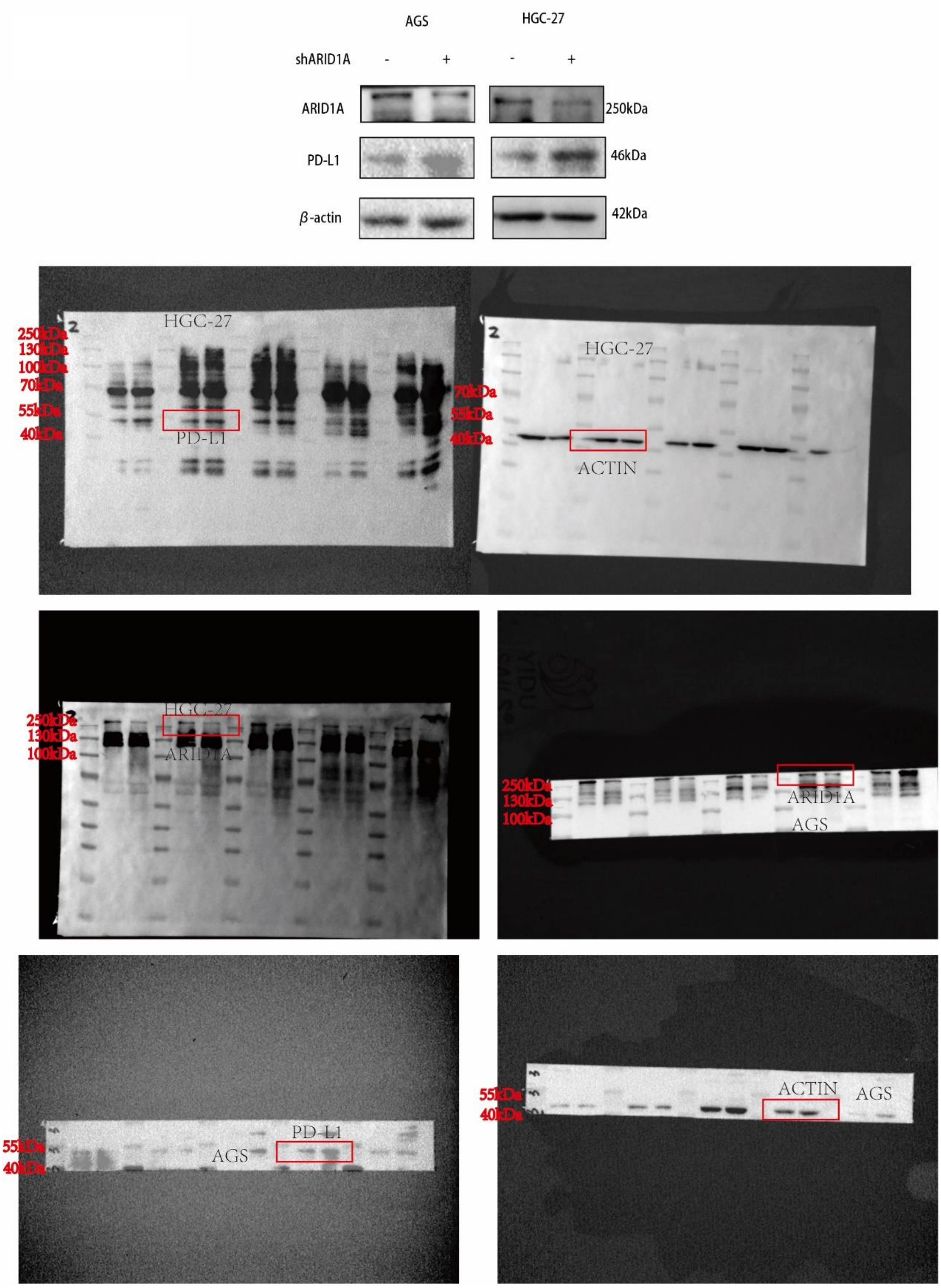

Figure10D

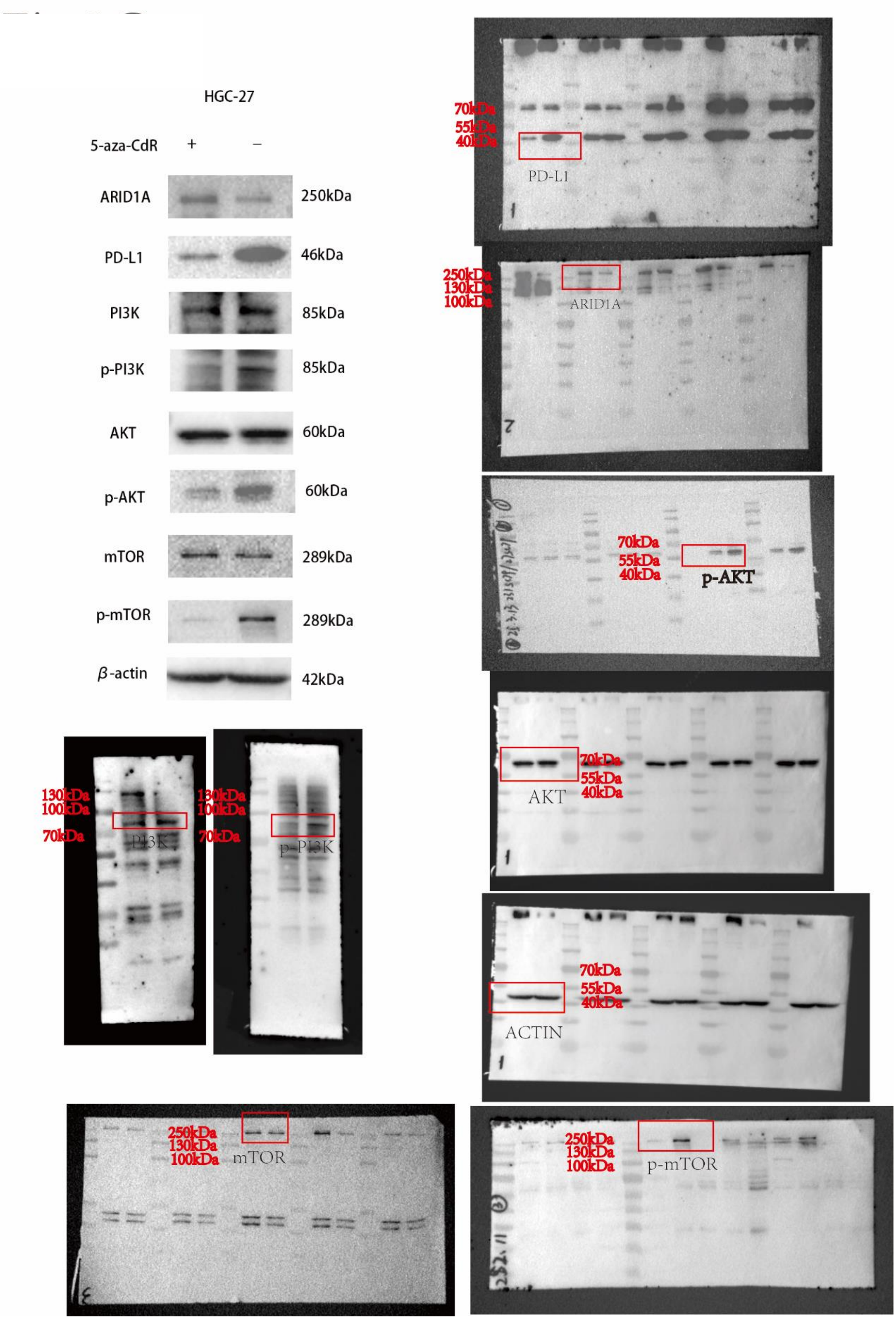

HGC-27
5-aza-CdR + −
ARID1A 250kDa
PD-L1 46kDa
PI3K 85kDa
p-PI3K 85kDa
AKT 60kDa
p-AKT 60kDa
mTOR 289kDa
p-mTOR 289kDa
β-actin 42kDa
70kDa
55kDa
40kDa
PD-L1
250kDa
130kDa
100kDa
ARID1A
p-AKT
AKT
ACTIN
PI3K
p-PI3K
mTOR
p-mTOR

Figure 10M

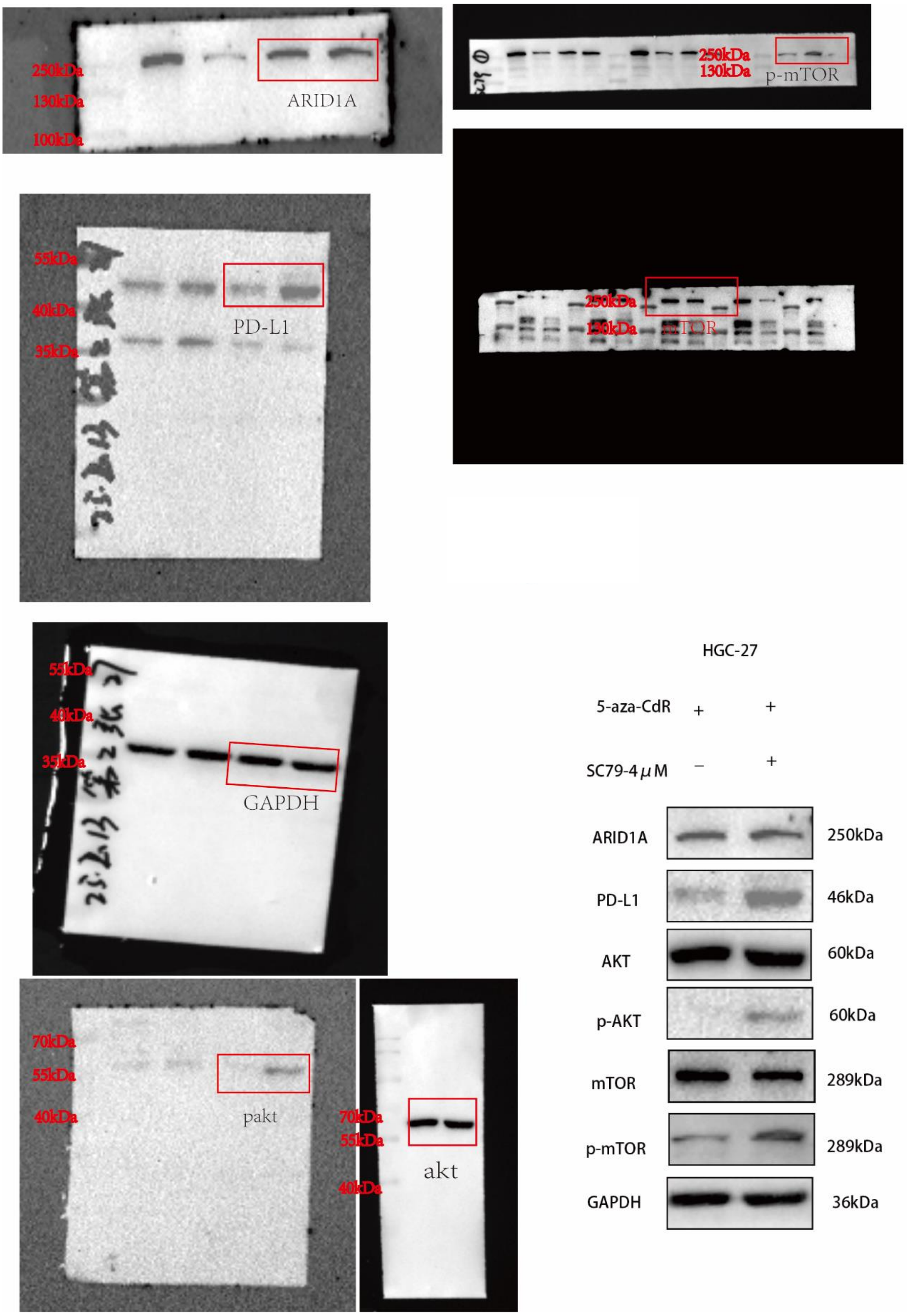

250kDa
130kDa
100kDa
ARID1A
250kDa
130kDa
p-mTOR
250kDa
130kDa
mTOR
55kDa
40kDa
35kDa
PD-L1
55kDa
40kDa
35kDa
GAPDH
70kDa
55kDa
40kDa
pakt
70kDa
55kDa
40kDa
akt
HGC-27
5-aza-CdR + +
SC79-4μM − +
ARID1A 250kDa
PD-L1 46kDa
AKT 60kDa
p-AKT 60kDa
mTOR 289kDa
p-mTOR 289kDa
GAPDH 36kDa

Figure10T

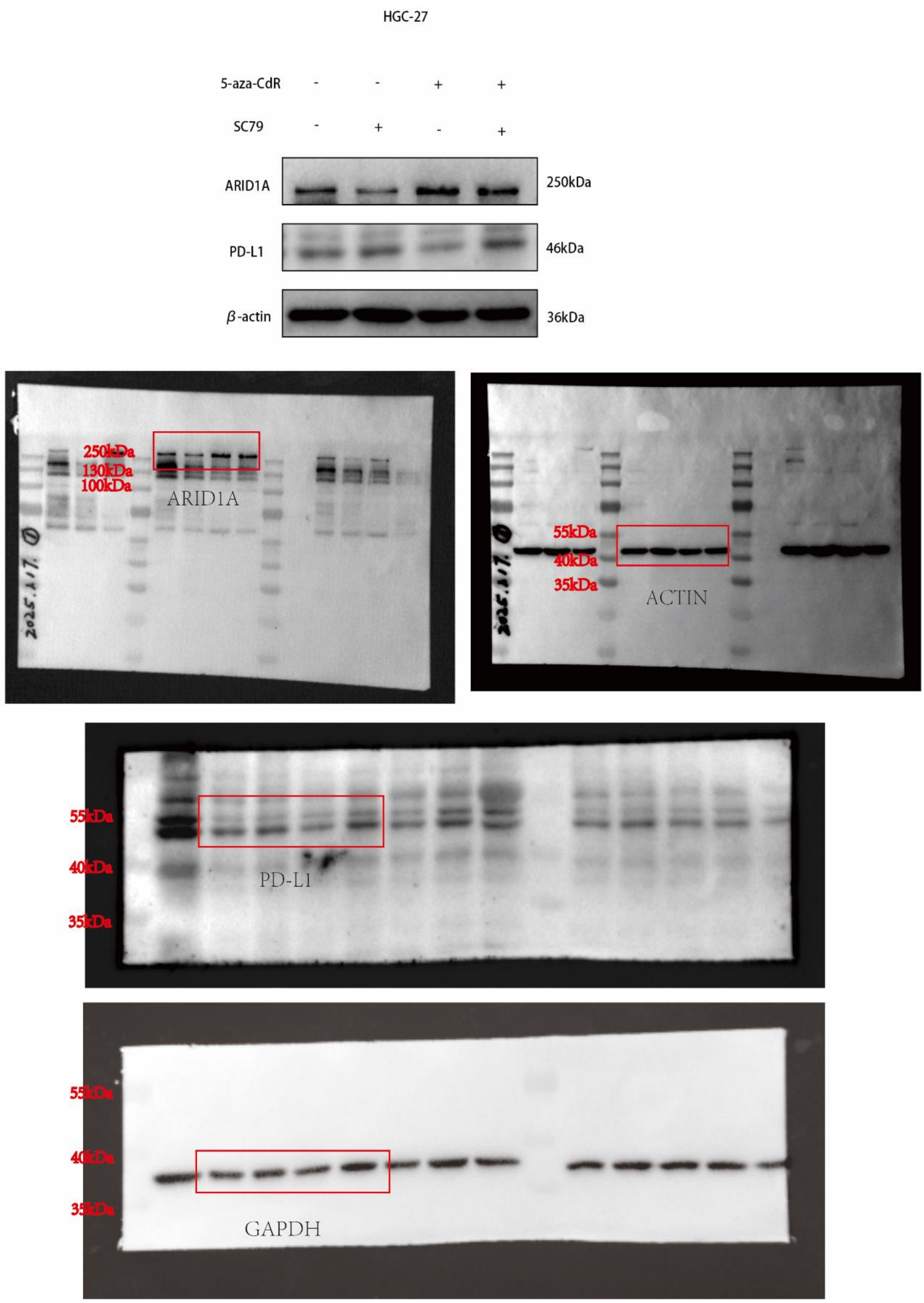
HGC-27
5-aza-CdR - - + +
SC79 - + - +
ARID1A
250kDa
PD-L1
46kDa
β-actin
36kDa
250kDa
130kDa
100kDa
ARID1A
55kDa
40kDa
35kDa
ACTIN
55kDa
40kDa
35kDa
PD-L1
55kDa
40kDa
35kDa
GAPDH

2.Original Images for Gels

Figure7B

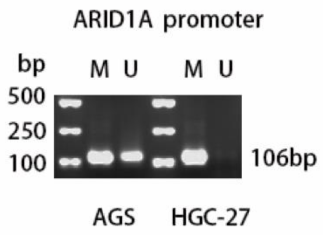

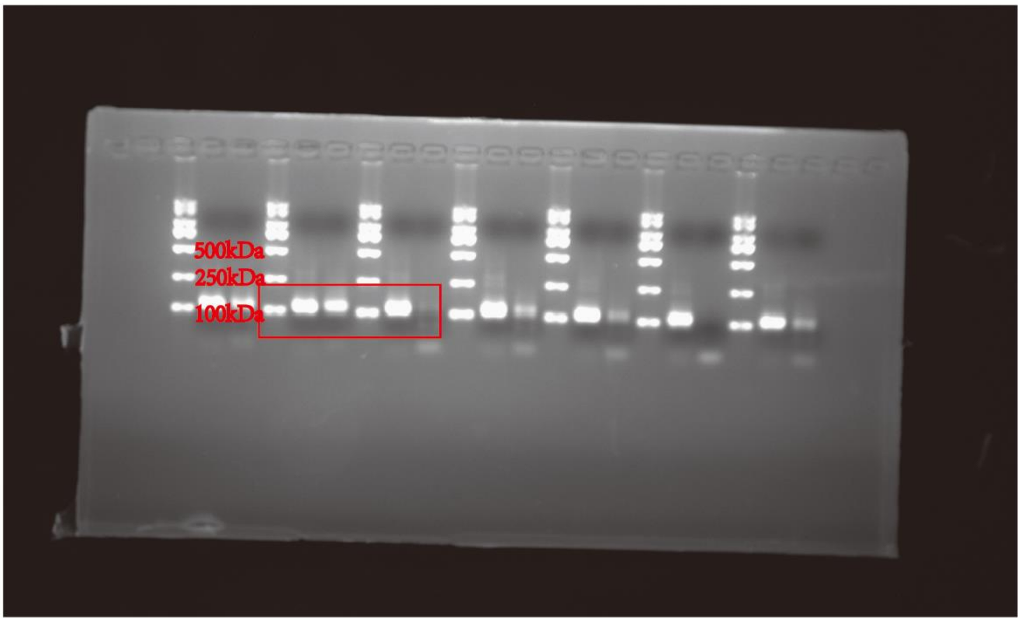

Figure7G

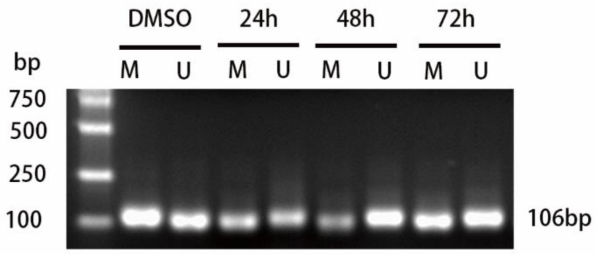

ARID1A promoter

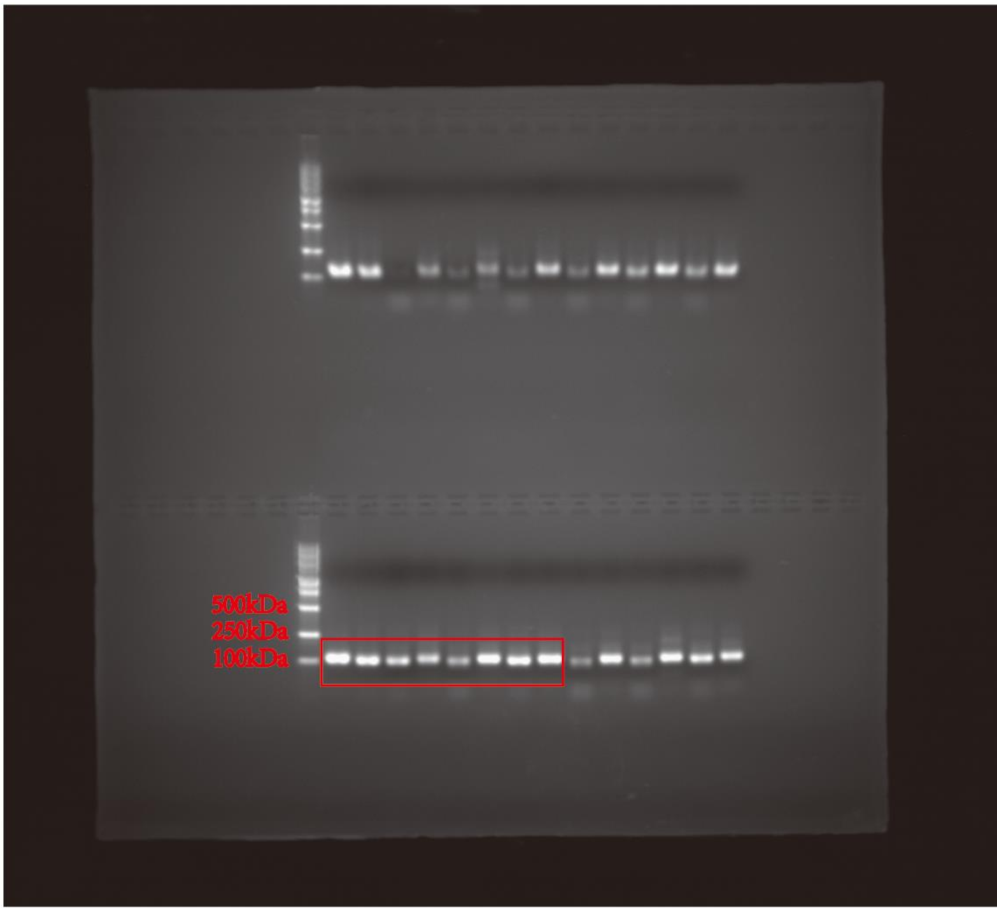

3.Original Images for Microscopy

Figure S5E

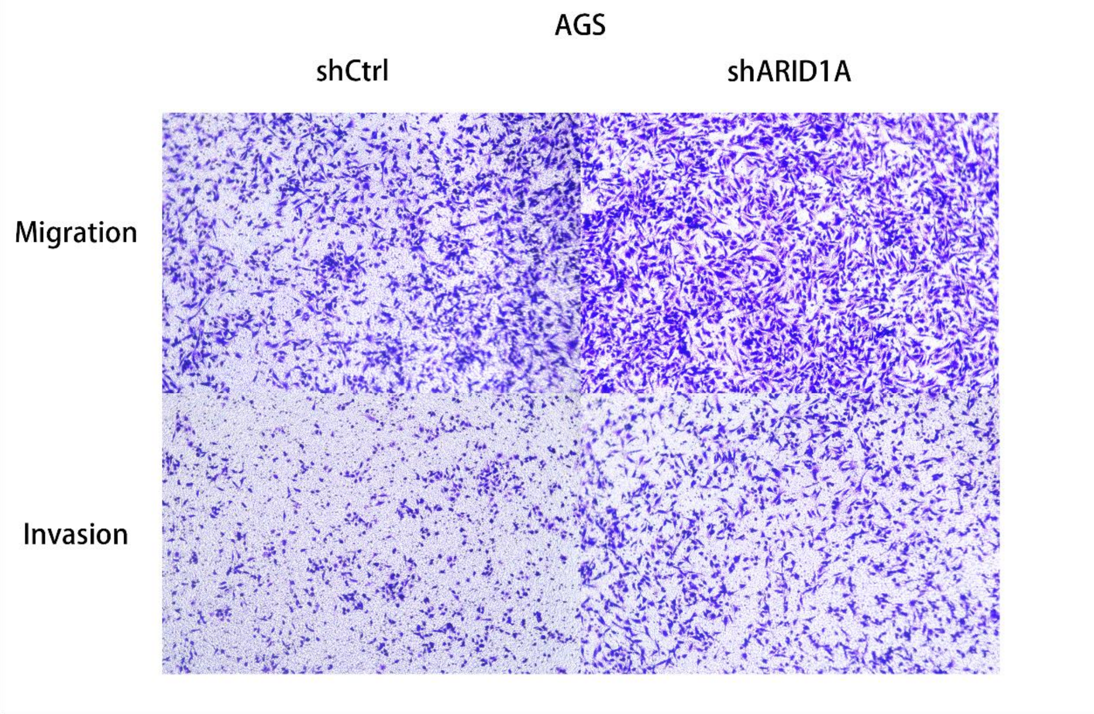

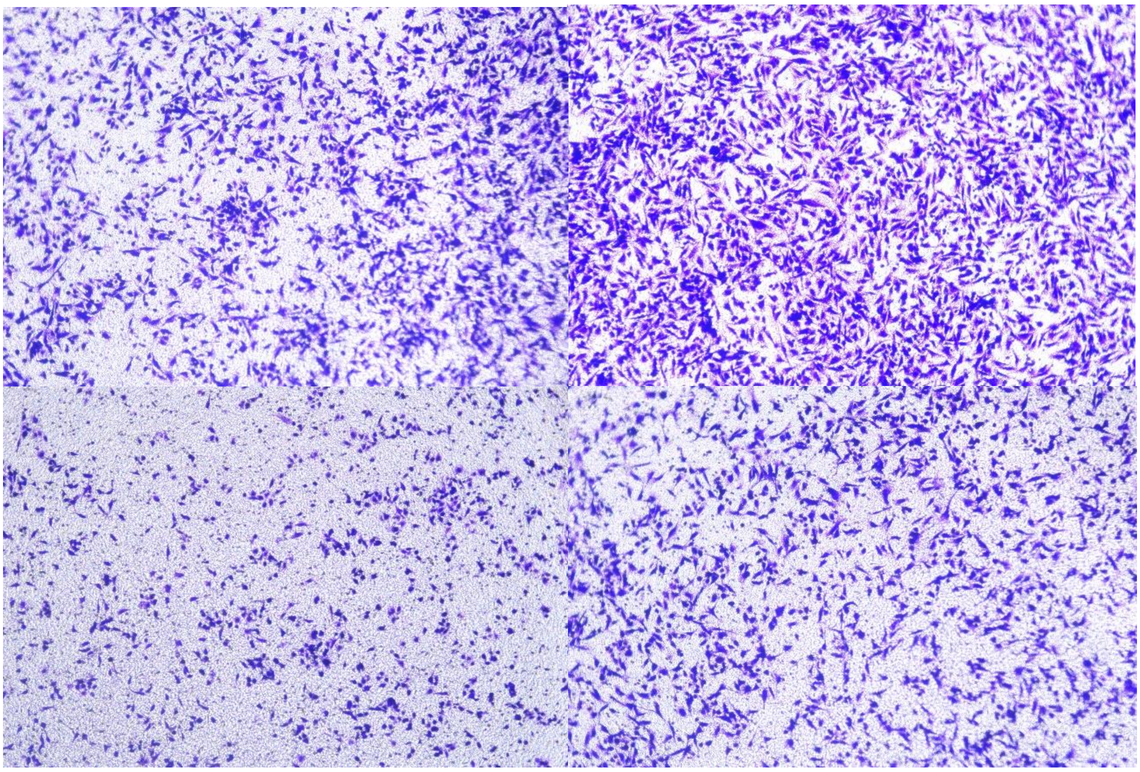

Fig-8E

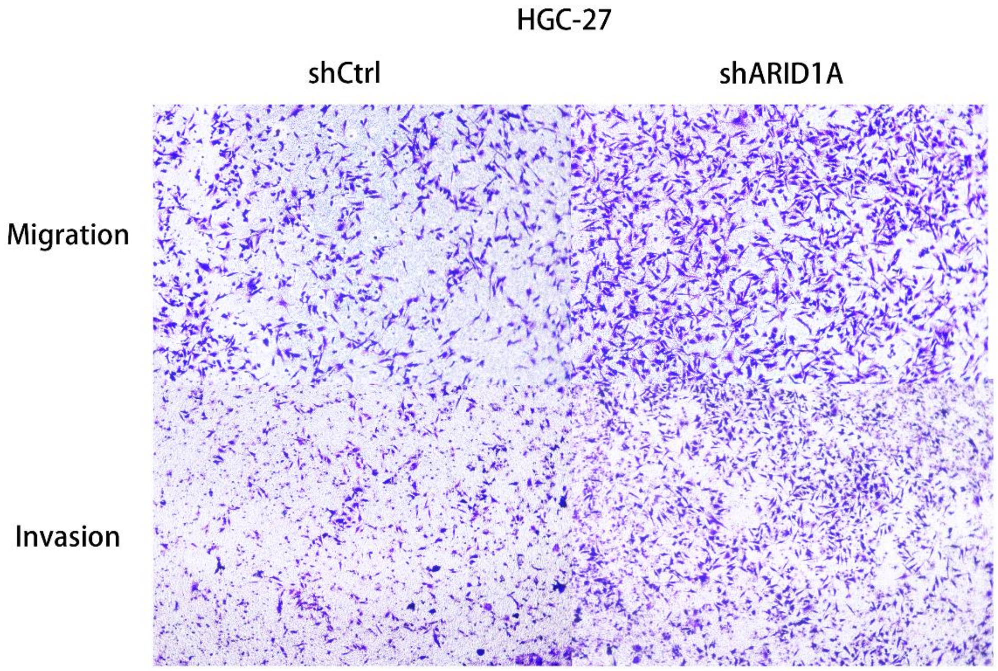

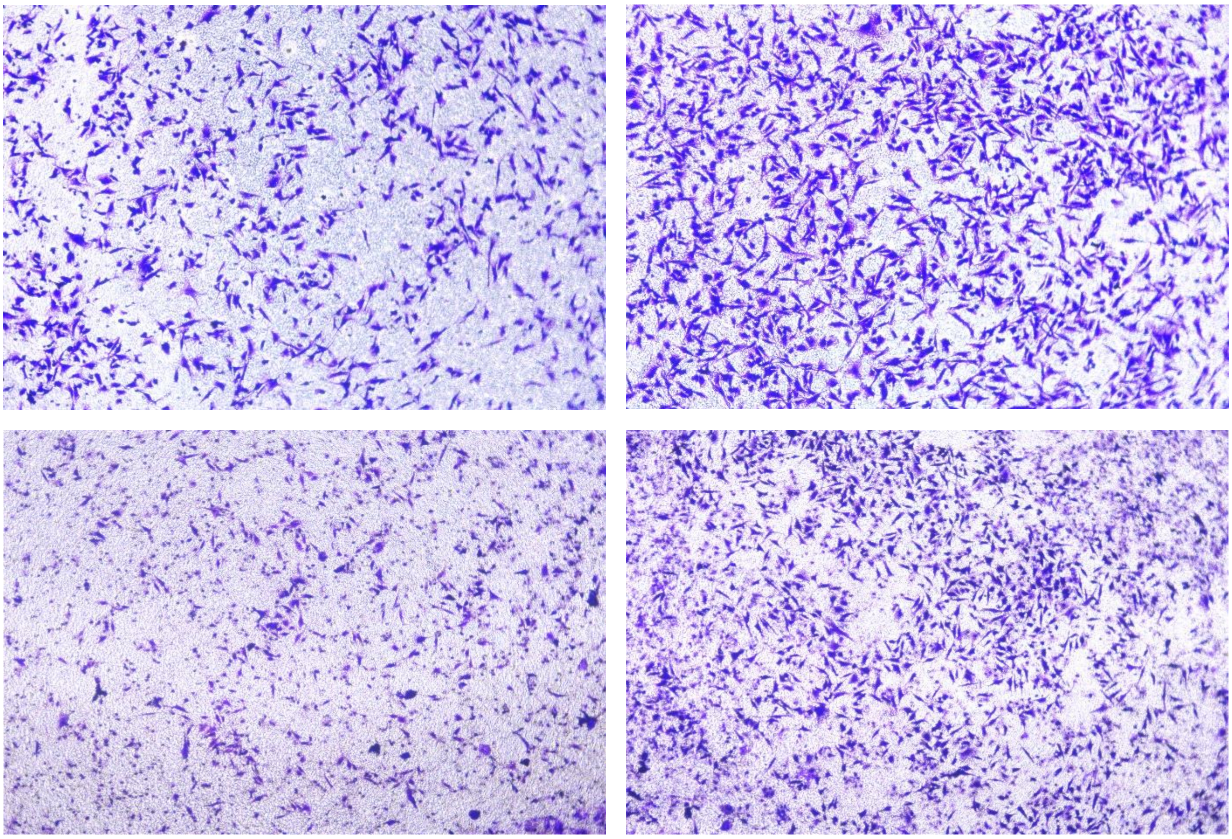

Figure S5H

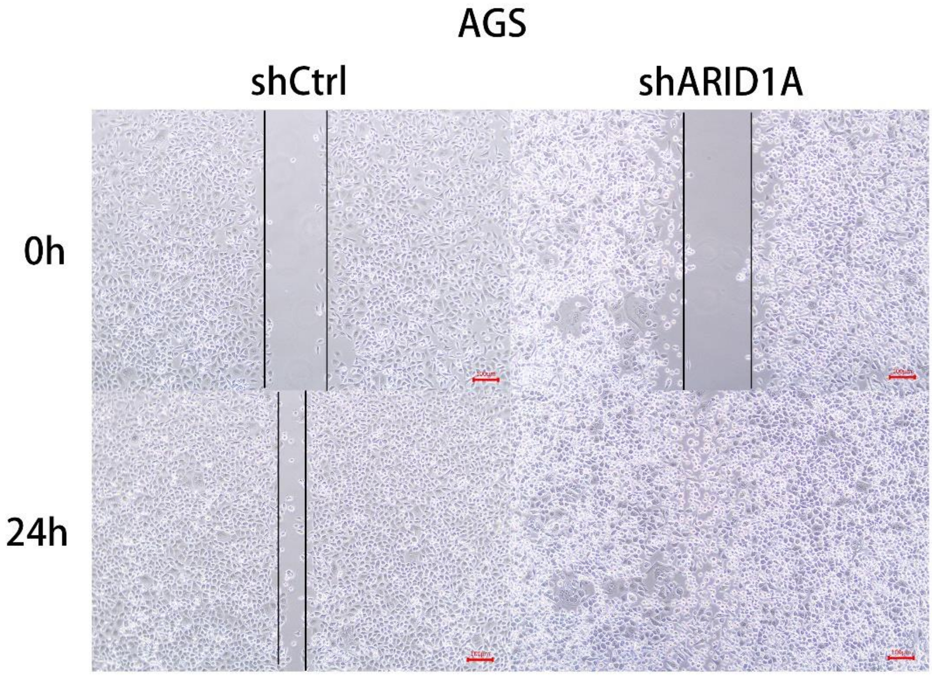

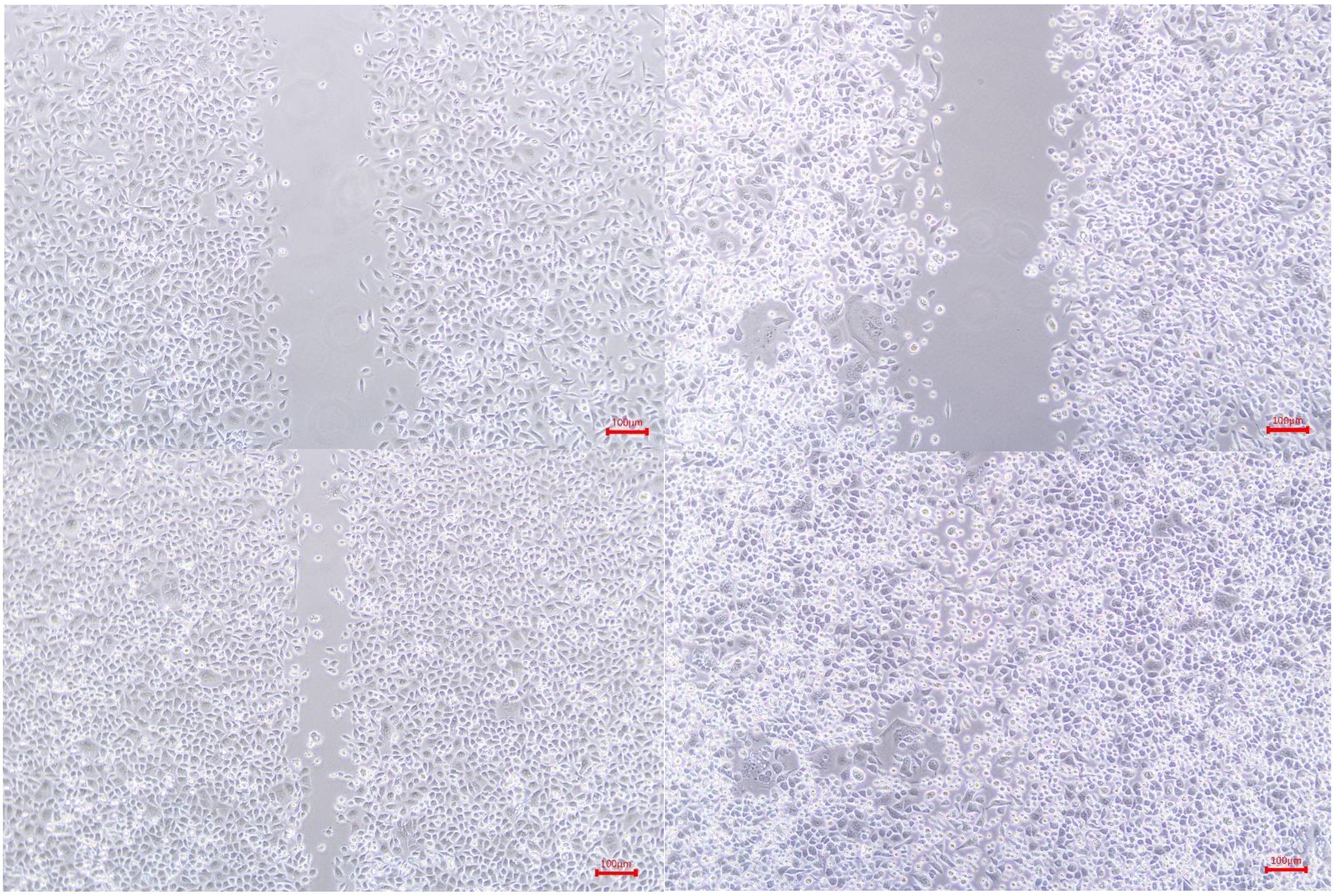

Figure 8H

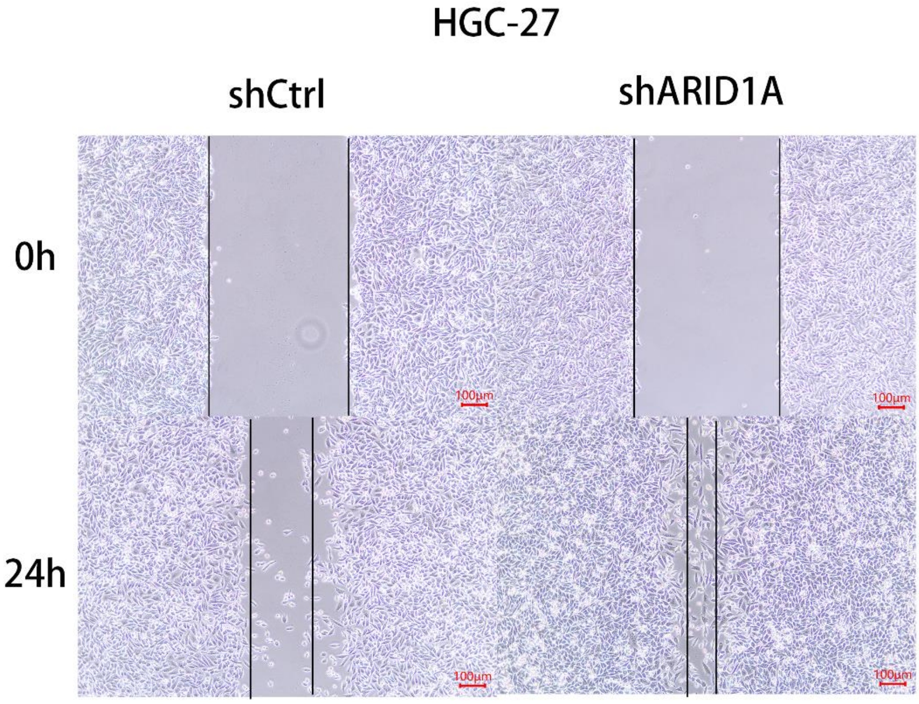
HGC-27
shCtrl
shARID1A
0h
24h
100μm
100μm
100μm
100μm

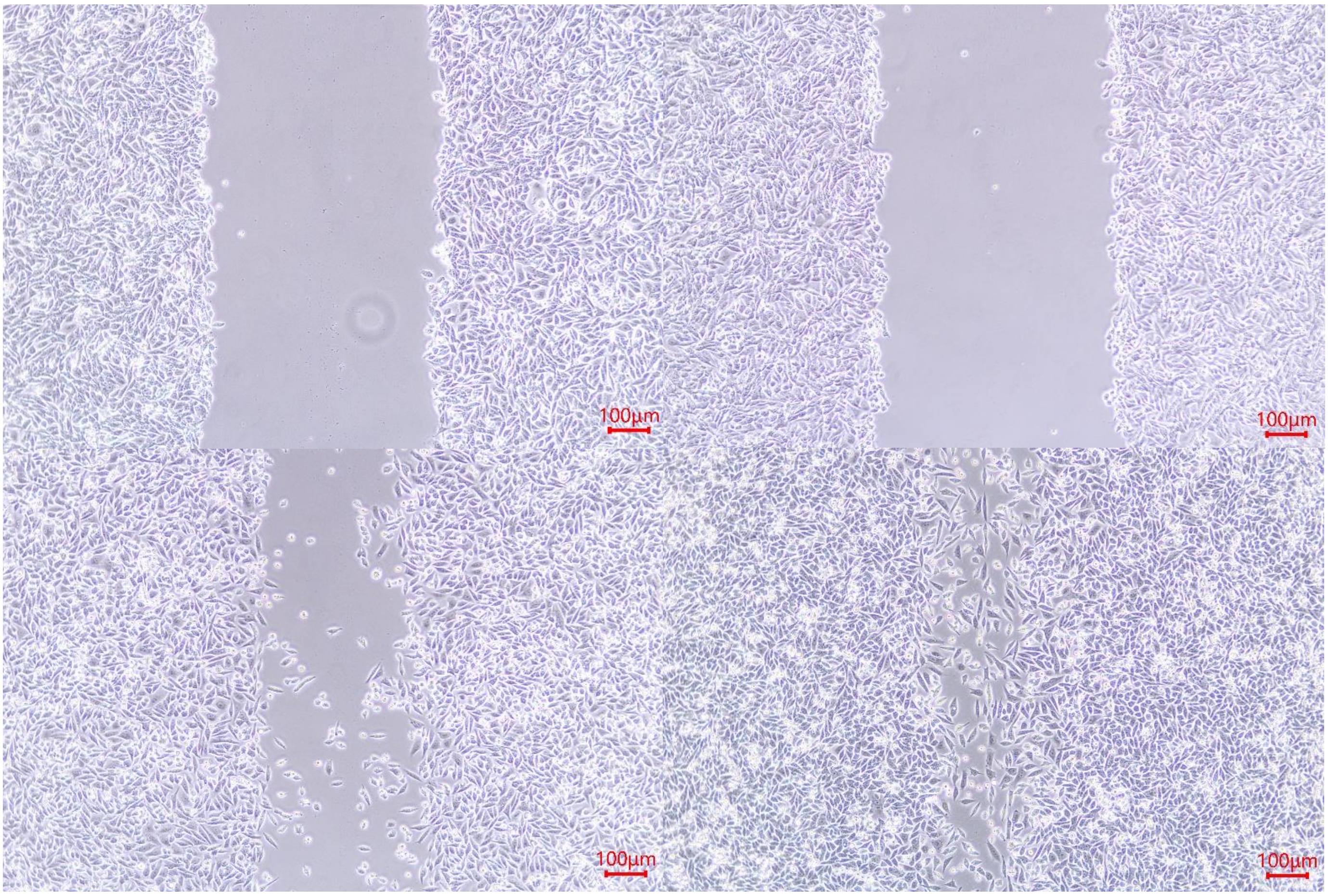
100μm
100μm
100μm
100μm

Figure S5I

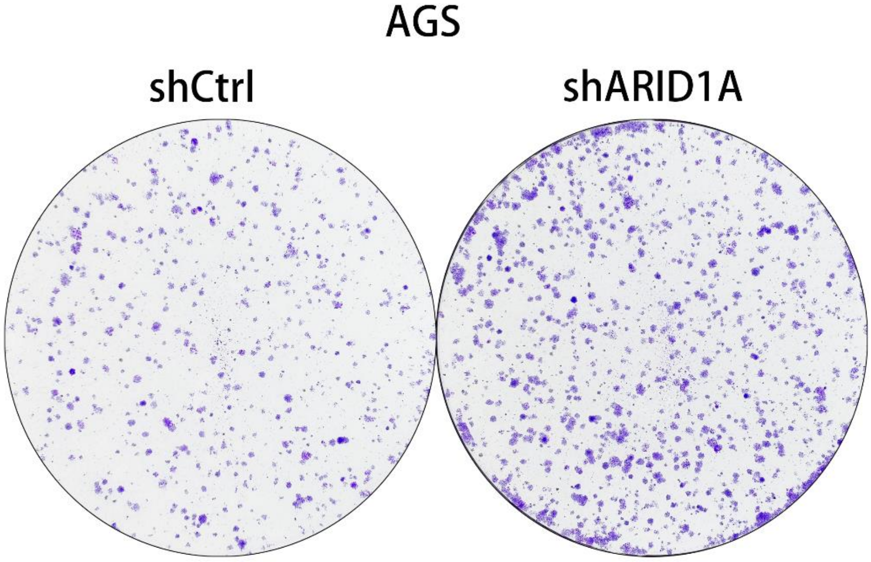

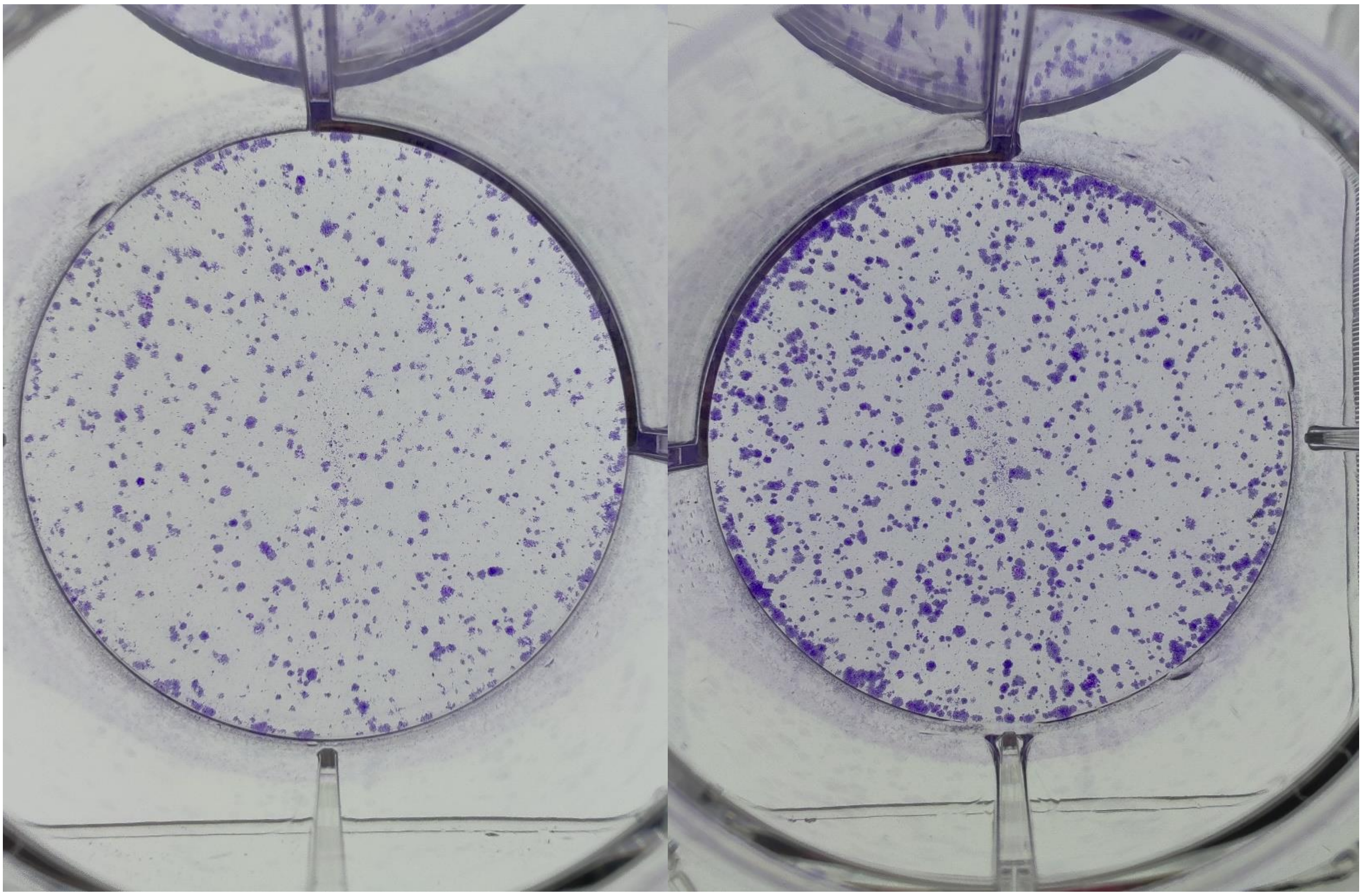

Figure 8I

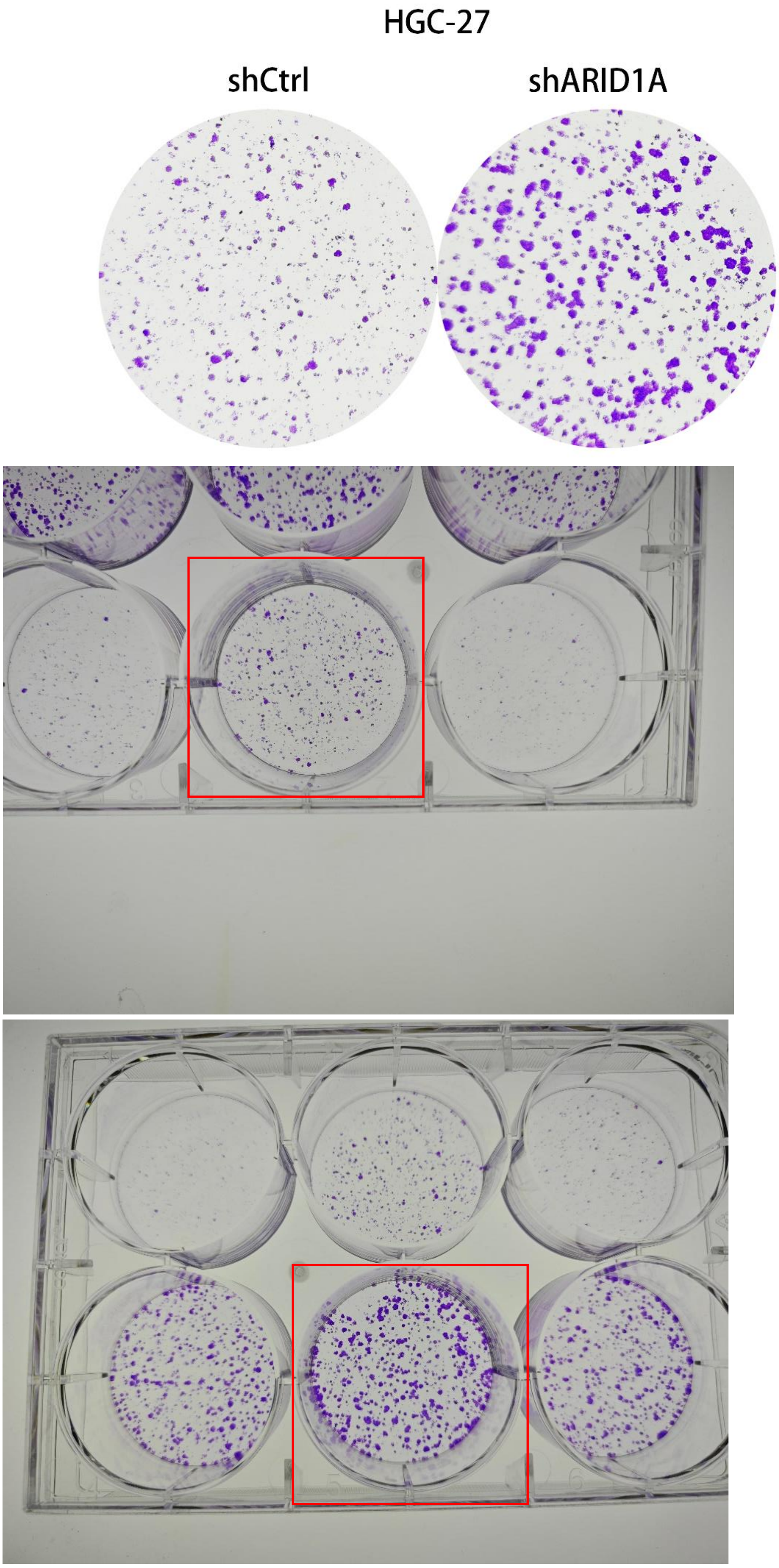

Figure 9A

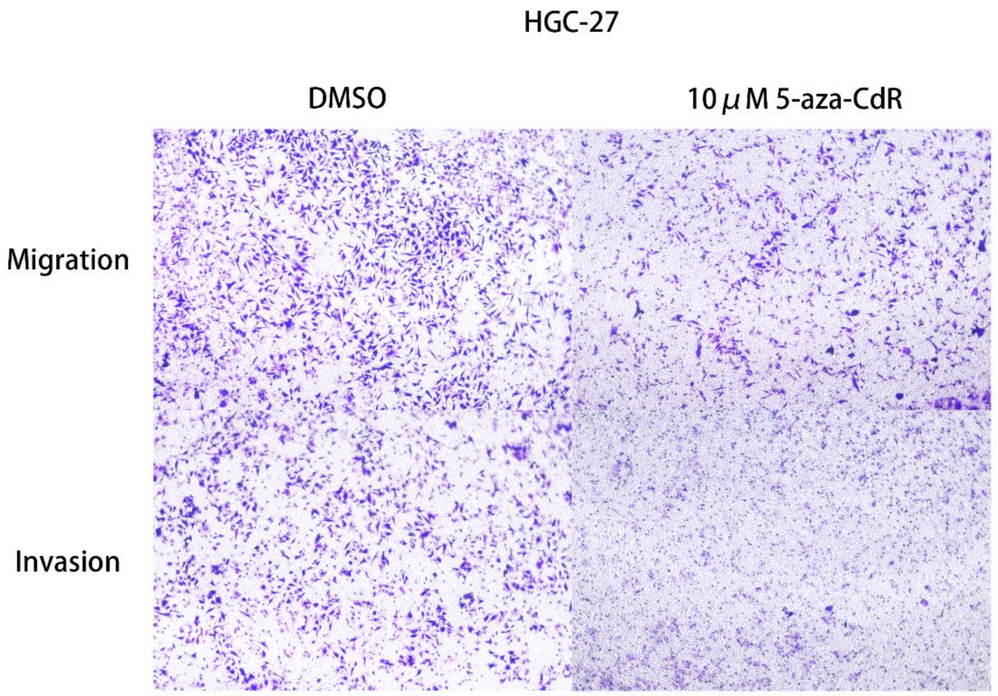

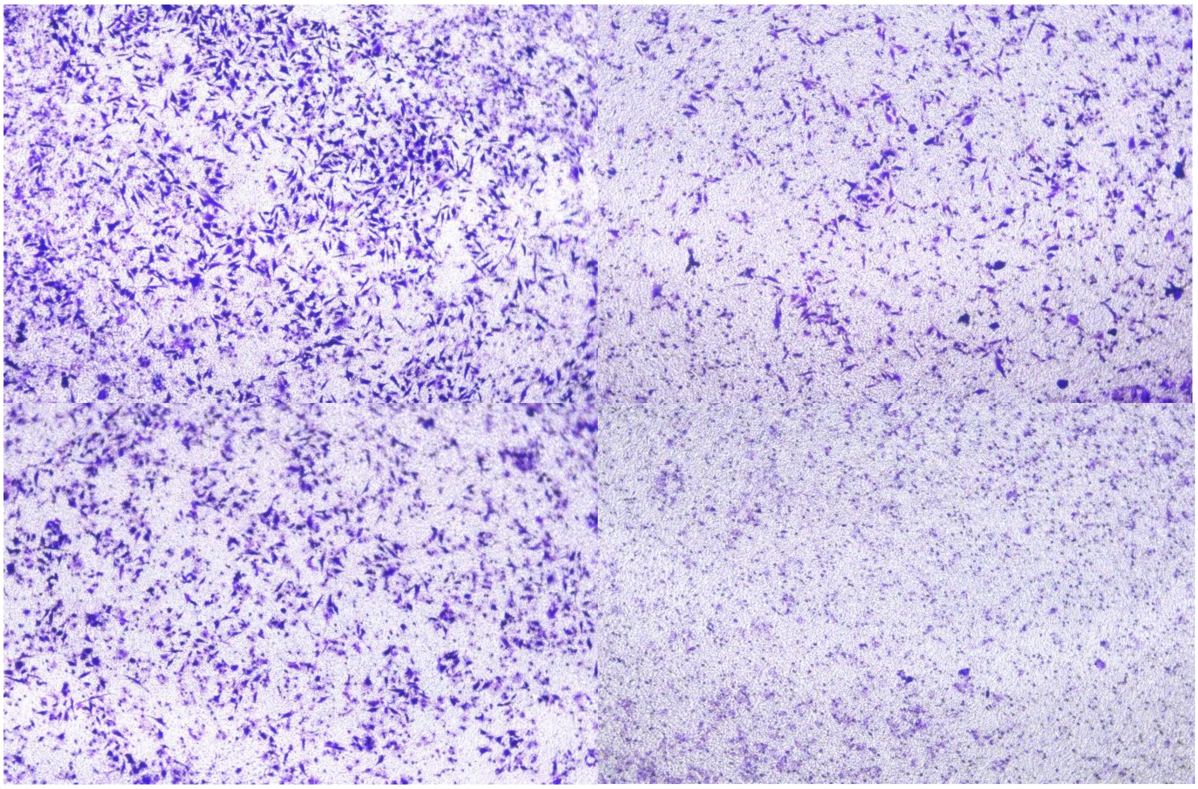

Figure 9D

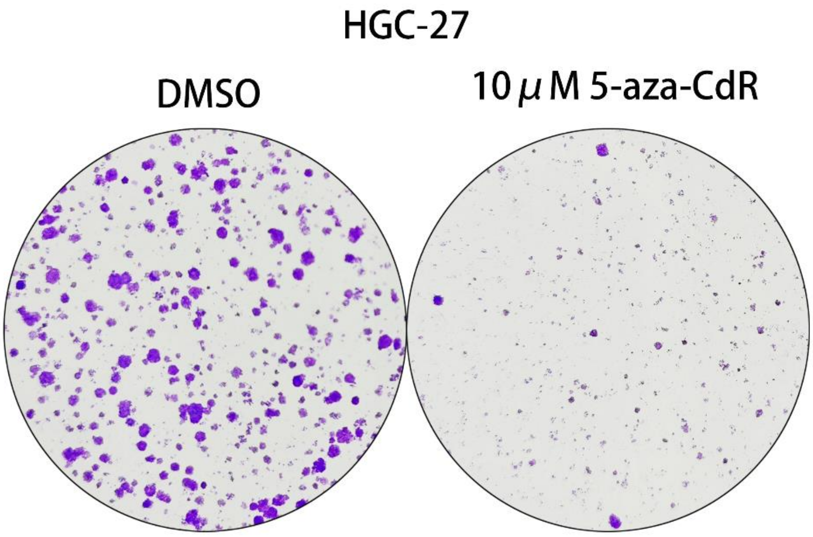

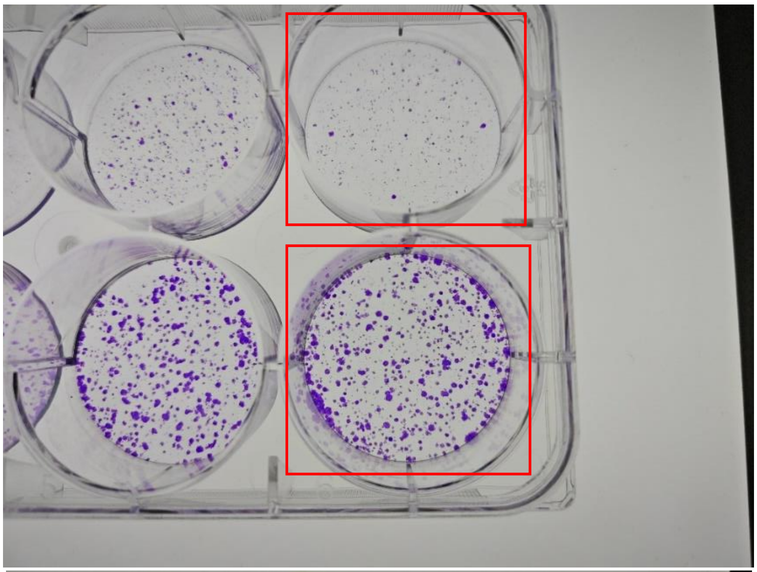

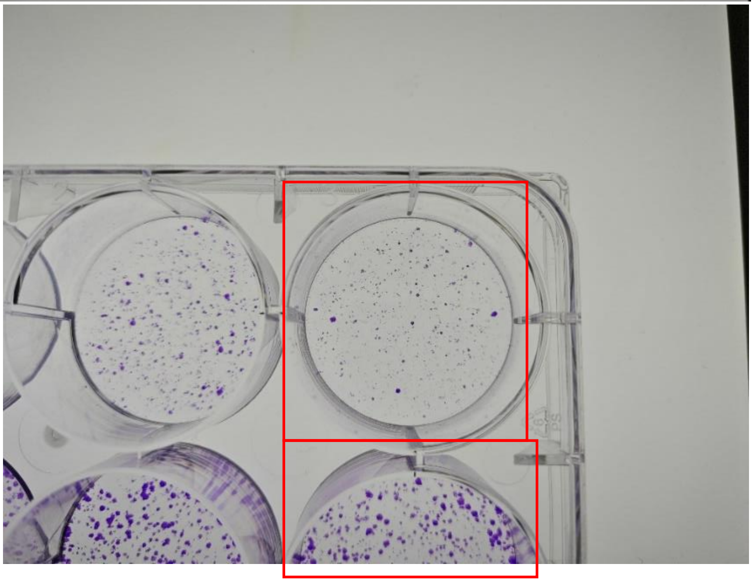

Supplement: Supplemental Information 8 [file peerj-13-20251-s008.pdf]
